# Why Do Patients With Ischaemic Heart Disease Drop Out From Cardiac Rehabilitation in Primary Health Settings. A Qualitative Audit of Patient Charts

**DOI:** 10.3389/fresc.2022.837174

**Published:** 2022-04-04

**Authors:** Maiken Bay Ravn, Maria Uhd, Marie Louise Svendsen, Lisbeth Ørtenblad, Thomas Maribo

**Affiliations:** ^1^Department of Public Health, Centre for Rehabilitation Research, Aarhus University, Aarhus, Denmark; ^2^DEFACTUM, Central Denmark Region, Aarhus, Denmark

**Keywords:** Cardiac Rehabilitation, qualitative study, audit, dropout, ischaemic heart disease, primary health settings

## Abstract

**Background:**

Cardiac rehabilitation (CR) and medical treatment are integrated parts of the intervention for cardiac patients and are a class 1A recommendation. However, CR dropout is reported to be relatively high and little is known about the reasons for CR dropout in primary health settings.

**Aim:**

This study investigates causes for CR dropout through a qualitative audit of medical charts among patients with ischaemic heart disease.

**Methods:**

This was a qualitative retrospective audit of patient's medical charts. Patients who dropped out from CR between 1 January and 31 December 2018 in five primary health settings were included. Local patient charts provided information related to causes and formed the basis of the analysis. Data were analyzed using thematic analysis.

**Results:**

A total of 690 patients were referred for and commenced CR and 199 (29%) dropped out. Twenty-five (12.6%) patients finished CR but were excluded due to standards of ≥180 days between CR meetings, leaving 118 patients included. Four themes as causes for patient's dropout were identified: (1) CR-programmes, (2) logistical, (3) intrapersonal and (4) clinical factors.

**Conclusion:**

This study identified new focus areas to which health professionals may attend in reducing drop-out from CR. Organisation of CR, challenges with combining labor market attachment and CR, focus on patient education and comorbidities. The results underline the importance of health professionals emphasizing the benefits of CR and explains that CR enhances long-term labor market attachment. Furthermore, health professionals should encourage participation in patient education and adapt exercise to the individual patient's potential.

## Introduction

Worldwide, ischaemic heart disease (IHD) is the most common cause of death (1). Cardiac rehabilitation (CR) and medical treatment are integrated parts of interventions for cardiac patients and a class 1A recommendation (2–4). CR is a multidisciplinary intervention encompassing core components targeting psychosocial and vocational support, lifestyle behavior changes, clinical stabilization and steps to reduce disability and risk factors (5). CR may reduce rehospitalisation, cardiac mortality and activity-related symptoms while improving functioning (6). CR is organized in a three-phased structure: phase I is the hospitalization period; phase II the period immediately after treatment at the hospital; and phase III the maintenance period (6, 7).

CR can be provided at hospitals or as an out-patient service at primary health settings. Low-risk patients are especially suitable for CR in primary health settings (5) where rehabilitation enables patients to remain in education or employment and reduces rehospitalisation (8). In the Central Denmark Region, CR programmes follow the European guidelines for CR, and low-risk patients with IHD are provided with phase II CR services in primary health settings (5, 8).

Despite knowledge about the positive effect of CR, drop-out rates are high, ranging from 17 to 39% (9, 10). CR dropout is associated with an increased risk of cardiovascular events and mortality (9). Factors related to dropout include gender, comorbidities, smoking status and exercise capacity (11). Even so, the patients' and health professionals' perspectives on dropout are poorly examined. Therefore, this study examined patient's causes for dropout from CR through a qualitative audit of the medical charts of patients with IHD. Another article explores health professional's perspectives on how to facilitate CR adherence (12).

## Method

### Qualitative Audit

This was a qualitative retrospective audit of patient's medical charts. Chart audits may be used for data collection in studies exploring clinical queries and patient adherence (13).

### Setting

The study was conducted in five primary health settings in the Central Region Denmark covering five municipalities with a total of 635,000 inhabitants, varying with respect to population size, population density and mix of urban and rural areas. The included settings all followed the guidelines on rehabilitation for patients with cardiac disease in which CR is a group-based intervention including several aspects; lifestyle, screening for anxiety and depression, return to work, psychosocial support and patient education (14).

### Eligibility Criteria

The qualitative audit included all IHD patients from the catchment area referred to CR from 1 January to 31 December 2018, and who commenced CR but dropped out. Two databases were used as information sources:

1. The Danish Database for Cardiac Rehabilitation in Primary Health Care Settings was used to identify dropouts. National quality standards were used. Information regarding CR enrolment, diagnosis and dropout was extracted from the database (15).
2. Local patient charts provided information related to causes. These charts contain open textboxes where health professionals write descriptions and observation notes regarding their patients. Furthermore, various types of communication (telephone, texts or e-mail) with the patients, hospital or GP are noted in the charts.

### Analysis

STATA was used to prepare the data, and NVivo 2.0 software was employed to organize data for the analysis. Data were analyzed using descriptive analysis inspired by Braun and Clark (16). The process included the following phases; (1) Reading the notes; (2) Generating initial codes; (3) Arranging codes into themes; (4) Discussing and reviewing codes and themes; (5) Final analysis and extraction of quotes for analysis (16).

Themes were created according to factors associated with dropout identified in a systematic review: intrapersonal factors, clinical factors, interpersonal factors, logistical factors, CR programme factors and health system factors (11).

### Patient Panel

A panel of former patients with heart disease was formed to validate the relevancy and transferability of our findings to practice. Patients were recruited through the Danish Heart Association. Due to the COVID-19 pandemic, a meeting was held online in which six patients participated. Initial results from the audit were presented and discussed with the panel. The perspectives of the patient panel were used to focus the analysis, thereby ensuring relevancy and transferability to future patients.

### Ethics

The project was approved by the Danish Patient Safety Authority (ID: 31-1522-28), and all involved primary health settings gave written consent.

## Results

**Figure 1:**
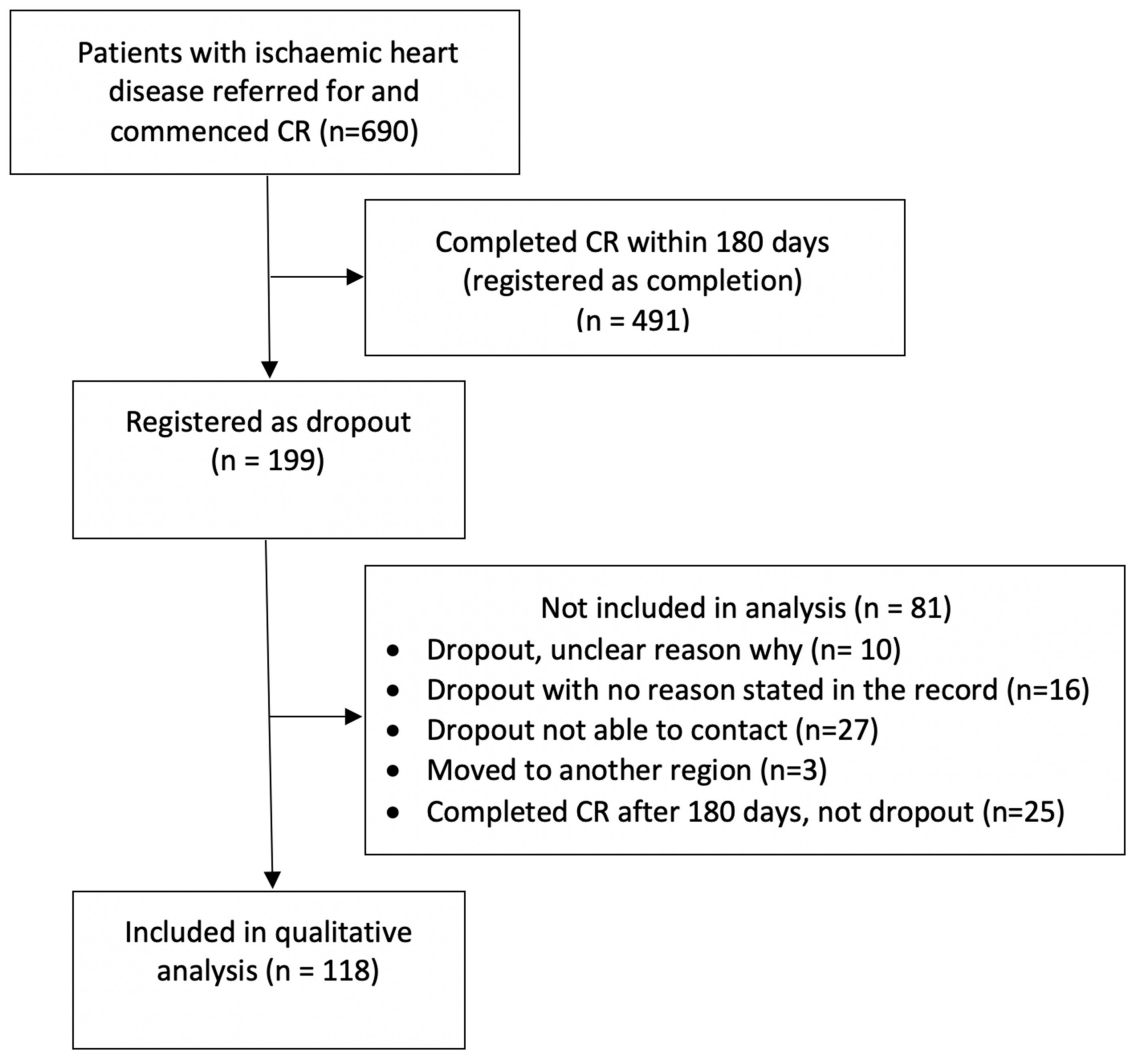
Flow diagram.

Among IHD patients, 690 were referred for and commenced CR in the period and 199 (29%) dropped out. Only patient charts in which a cause for dropout could be identified were included in the analysis. The charts of 16 patients were unclear, ten dropped out without reasons being provided, 27 dropped out without contact to the primary health setting and three moved out from the primary health setting. The standards of the Danish Database for Cardiac Rehabilitation in Primary Health Care provided that patients having ≥180 days between their initial and final CR meeting were recorded as dropouts. However, 25 patients finished CR ≥ 180 days after a CR meeting but were not dropouts, leaving 118 patient charts for inclusion in the qualitative analysis, see Figure 1. Of the 118 included patient charts, 96 patients (81.4 %) were male and the mean age were 65 years ranging from 38 to 88 years.

The number of notes in the patient charts varied from one for patients who dropped out early in CR to 47 notes. After reading and coding notes of all 118 patients, 17 codes were created and divided into four themes based on the systematic review: CR programmes, logistical, intrapersonal and clinical (11). A total of 133 causes for drop out were identified. Table 1 provides an overview and distribution of the overarching themes and the codes.

**Table 1:** Themes and codes from the audit.

**Theme**	**Codes**
CR programme (*N* = 44)	Self-administrating training
	Not feeling any gain from CR
	Former experience with rehabilitation
	Unsatisfied with CR
Logistical (*N* = 20)	Planning
	Work
	Transportation
Intrapersonal (*N* = 41)	Not feeling the need
	Medicalisation
	Psychological difficulties
	Discomfort doing exercise
	Lack of energy and drive
Clinical (*N* = 28)	Side-effects from medication
	Comorbidity
	Cardiac complications
	Advised against doing exercise
	Specialized rehabilitation

### CR Programme

Preferring to administer training oneself was the reason most frequently stated in the notes related to the CR programme. According to the health professional's notes, some patients wanted to exercise in a regular gym, whereas others preferred a local center near their home or to exercise at home.

“*The patient wants to end CR and exercise by himself. Informs that he is working out in a gym. Is offered a meeting with a physiotherapist but refuses. Offered a watt-max test after 12 weeks of exercise by himself, but refuses” (Telephone conversation with a patient)*.

The reasons for this preference among patients were unclear from the audit. A few notes suggested that patients felt no benefit from CR. All these reasons were related to exercise. Why patients did not attend patient education or other parts of CR remains unclear from the notes. According to the health professional's notes, a few patients had experience with CR from a previous cardiac event and argued feeling well-informed about how to handle their cardiac disease. This was related to exercise and patient education alike. Notes from one patient expressed direct dissatisfaction with the CR programme as the reason for dropout.

### Logistical Factors

Planning was related to both the wish for more flexible hours in the CR programme and private-life planning. Having travel plans was another drop-out reason that appeared in several notes. This was evident in the health professional's notes and in e-mail or text communications with patients.

Challenges associated with patient's employers were also a CR drop-out reason. Some patients felt pressured to return to work quickly or found that it was overwhelming to attend CR while returning to work.

“*Generally, I can't manage anything except my job and my check-ups at the hospital right now where I attend check-ups regarding hypertension. My employer is not very helpful at the moment”* (E-mail from a patient).

As this patient's e-mail shows, uncertainties relating to job situation placed the patients under pressure and, for some patients, this triggered CR dropout.

In three notes, transport to and from CR was stated as the reason for dropout. Feeling discomfort when sitting in a car, having difficulties walking to and from the facility and financial issues were additional reasons mentioned in the health professional's notes.

### Intrapersonal Factors

Not feeling the need for CR was identified in many different forms in the notes, and not feeling sick or experiencing any cardiovascular-related discomfort were the most often stated reasons.

“*The patient wishes to end the CR programme. He feels he is doing well, has no discomfort related to his heart and does not feel any limitations in his life. He does not feel he is sick in any way”* (Note from a health professional).

For these patients, CR was perceived as part of a treatment for people who are sick and, as they did not experience being sick, they were not interested in attending CR. In the health professional's notes, one patient who dropped out of CR argued feeling more sick when attending CR.

Coping with psychological difficulties was another intrapersonal factor related to dropout. Stress related to work, health and personal life triggered dropout for some. Anxiety and depression were the main barrier for some patients. Frequently, the health professional's course of action was to consult with the patient's general practitioner. However, dropout was not always prevented.

Discomfort during exercise was also an intrapersonal factor related to dropout. Delayed-onset muscle soreness after high-intensity work or training was a barrier for some patients. Some of these patients were unfamiliar with exercising and found that the exercise was too advanced for them. Knowledge regarding the benefits and importance in exercising after a cardiac event was, however, not lacking; so for some this was an internal struggle.

“*The patient is concerned that the pain in his legs will intensify when doing exercise and that effects his motivation to continue. He is, however, concerned for his health if he does not start doing exercise” (Note from a health professional)*.

For some patients, a lack of energy and drive was also related to an internal struggle.

“*The CR programme seems chaotic for her right now. On one hand, she believes that doing exercise might help her, but on the other hand, it is completely overpowering and she already knows that she will not be able to motivate herself to attending” (Note from a health professional)*.

### Clinical Factors

Experiencing side-effects from their medication was a factor influencing CR dropout. The side-effects were mostly related to a lack of energy and feeling tired after exercising. Several patients had comorbidities influencing their ability to attend CR. Musculoskeletal disorders and previous sport injuries were a barrier experienced by some patients. These patients were not being advised against exercising and therefore, dropping out was their own decision.

“*The patient informs us that he wants to drop out as he experiences complications with an already familiar issue in his knee. He is offered a tailored exercise programme but does not want this”* (Note from a health professional).

Dropout was also associated with cardiac complications. For some patients, cardiac complications caused them to place CR on standby for a period and then resume CR later. Hence, these patients did not drop out, but they did complete >180 days after the initial meeting, whereas other patients terminated CR. Some of the patients experiencing cardiac complications are, however, advised against exercising.

All of these factors were related to exercise exclusively and not to the CR programme as a whole. The reasons why these patients did not attend the remaining parts of the CR programme remained unclear from the notes. A few patients dropped out of CR to attend specialized CR at the hospital.

## Discussion

This study provided knowledge about IHD patient's dropout causes. We included 118 patients and analyzed their medical charts. Patients who dropped out did not differ from those who completed CR with respect to sex and age.

Four main causes for CR dropout in the primary health settings were established; CR programmes, logistical, intrapersonal and clinical. Although the themes were based on a systematic review, the results from this audit provide a more in-depth understanding of the causes for CR dropout (11). Two factors from the systematic review-*Interpersonal factors* and *Health system factors*-were not identified in the audit (11). Interpersonal factors related to dropout identified in the review were being single, unemployed or retired. The health system factor related to dropout was a longer interval between the first and the second visit (11). Neither of these causes was identified in the audit. However, the patient's civil status and employment status were not included in the audit. Furthermore, none of the notes included causes related to duration and timing of visits.

According to this audit, the structure and organization of CR programmes as a cause for dropout were related mainly to the exercise aspect of CR. However, CR includes more than exercise (2, 5). Studies indicate that patient education in CR may improve health-related quality of life (HRQoL) and reduce fatal and/or non-fatal cardiovascular events (17). The patient panel in this study also highlighted that patient education was an important part of their treatment and recommended encouraging patients to participating. Furthermore, studies have shown that managing psychosocial issues like stress tailored to the individual's needs improved patient outcome (18). It was not possible from the medical notes to evaluate how health professionals engage with and motivate patients; but health professionals should encourage patients to participate in patient education and highlight their benefits from doing so.

Among the logistical causes, planning CR was a predominant reason for dropout. Scheduled travels, lack of flexibility and work obligations were concerns relating to CR planning. According to the health professional's notes, they tailored the CR programmes to fit the patient's everyday life. Furthermore, it is clear from the audit that health professionals supported patients and counseled them in relation to their employer. Even so, some patients prioritized returning to work and argued that they were unable to find the time for CR. A 1-year study found that, despite a rapid return to work after a cardiac event one in four patients was detached from employment (19). Stressing the importance of CR in relation to return to work in the long run should therefore be highlighted and used as a motivation aid in relation to these patients.

Traveling time to and from the CR site is a known cause for CR dropout (20, 21). However, in this audit, traveling time was not a frequently identified drop-out cause. In Denmark, CR is integrated into the primary health settings as an initiative to provide healthcare close to the patient's homes (14). Providing CR closer to patient's home may have reduced traveling time as a drop-out cause in the included primary health settings.

Patient's perceptions of their disease is a well-known barrier to CR and one of the causes for dropout (18). Patient's perception of their disease changes over time and moves from a more acute view in the first week to a more chronic stage after 4 months (22). To accommodate these changes over time, health professionals may consider implementing a follow-up after a few months and invite dropouts to initiate a new CR programme.

A lack of energy and feeling too tired to participate in CR was an identified cause for dropout. Fatigue and lack of energy are common in patients with cardiac disease, and studies have shown that these symptoms are related to anxiety and depression. Furthermore, fatigue and lack of energy are predictors of a low HRQoL (23, 24). However, participation in CR is associated with an increase in energy and a reduction of fatigue in patients (25, 26). Health professionals should therefore stress that CR may help reduce fatigue and lack of energy when faced with patients who consider dropping out.

This audit provides several causes for dropout from CR. Patient's perception of illness, psychosocial factors, doing exercise at home or at another center are causes identified in previous studies (18, 27, 28). This audit, however, provided new insight into these causes. Some of the causes are modifiable and should be addressed by the health professionals in their work to motivate people to engage in CR. The patient charts clearly show that health professionals sought to accommodate individual preferences. However, it is unclear how they work with motivating patients; hence, this is being further investigated in another study (12).

The present study has a number of strengths and limitations. One of the strengths is the volume of data material. Notes from all patient dropouts in five primary health settings in the course of one year were included. The five primary health settings presented variation with respect to geography and population size, population density and mix of urban and rural areas.

This study also has some limitations. The audit was based on notes recorded by health professionals. The reasons for CR dropout are broadly the health professional's interpretation although some are the patient's own words as stated in written communication with the health professionals.

This study is based on data from a region in Denmark where CR is placed in the primary health settings. Placing a larger responsibility on care has increased in recent years and several countries offer CR in the primary health settings (29). The results from this study may be transferred to countries with CR in primary health settings or other similar settings.

In conclusion, the results identify new focus areas that health professionals may attend when facing patients who consider CR dropout:

- For barriers within the CR-programme, health professionals should be aware of patients who want to exercise elsewhere and highlight the benefits of participation in patient education.
- For logistical barriers, health professionals should pay attention and provide information for patients who are still working regarding the importance of CR in relation to return to work in the long run. This information should be given to the patient and their employer. The flexibility of CR programmes could also be adjusted.
- For barriers related to intrapersonal factors, health professionals should pay attention to patients who are unfamiliar with exercise, and tailor exercise to the patient including information on possible muscle soreness. Furthermore, highlight the benefits of participation in patient education.
- For barriers related to clinical factors, health professionals should focus on patients with comorbidities, and plan tailored exercise according to the patient's comorbidity. Furthermore, health professionals should address that fatigue is normal after a cardiac event and highlight that participating in CR may reduce this.

The health professional's perspectives on adaptation to individual patient needs are explored in another study (12). However, exploring the underlying reasons for patients preferring to exercise elsewhere is unclear in this study and further studies exploring this are needed. Furthermore, planning tailored exercise in relation to specific comorbidities in daily practice also needs to be further explored.

## Data Availability Statement

The original contributions presented in the study are included in the article/supplementary materials, further inquiries can be directed to the corresponding author.

## Author Contributions

MR drafted the manuscript. All authors contributed to the design of the work, analysis of data, revised the manuscript critically and gave final approval, and accept being accountable for all aspects of the work ensuring its integrity and accuracy.

## Funding

This project was funded by Public Health in the Central Denmark Region–and was a collaborative effort including municipalities as well as the Region, Grant No. A2617.

## Conflict of Interest

The authors declare that the research was conducted in the absence of any commercial or financial relationships that could be construed as a potential conflict of interest.

## Publisher's Note

All claims expressed in this article are solely those of the authors and do not necessarily represent those of their affiliated organizations, or those of the publisher, the editors and the reviewers. Any product that may be evaluated in this article, or claim that may be made by its manufacturer, is not guaranteed or endorsed by the publisher.
